# Acidosis Maintains the Function of Brain Mitochondria in Hypoxia-Tolerant Triplefin Fish: A Strategy to Survive Acute Hypoxic Exposure?

**DOI:** 10.3389/fphys.2018.01941

**Published:** 2019-01-18

**Authors:** Jules B. L. Devaux, Christopher P. Hedges, Nigel Birch, Neill Herbert, Gillian M. C. Renshaw, Anthony J. R. Hickey

**Affiliations:** ^1^School of Biological Sciences, The University of Auckland, Auckland, New Zealand; ^2^Institute of Marine Science, The University Auckland, Auckland, New Zealand; ^3^School of Allied Health Sciences, Griffith University, Gold Coast, QLD, Australia

**Keywords:** pH, hypoxia tolerance, mitochondria, lactate, acidosis, brain

## Abstract

The vertebrate brain is generally very sensitive to acidosis, so a hypoxia-induced decrease in pH is likely to have an effect on brain mitochondria (*mt*). Mitochondrial respiration (JO_2_) is required to generate an electrical gradient (ΔΨm) and a pH gradient to power ATP synthesis, yet the impact of pH modulation on brain *mt* function remains largely unexplored. As intertidal fishes within rock pools routinely experience hypoxia and reoxygenation, they would most likely experience changes in cellular pH. We hence compared four New Zealand triplefin fish species ranging from intertidal hypoxia-tolerant species (HTS) to subtidal hypoxia-sensitive species (HSS). We predicted that HTS would tolerate acidosis better than HSS in terms of sustaining *mt* structure and function. Using respirometers coupled to fluorimeters and pH electrodes, we titrated lactic-acid to decrease the pH of the media, and simultaneously recorded JO_2_, ΔΨm, and H^+^ buffering capacities within permeabilized brain and swelling of *mt* isolated from non-permeabilized brains. We then measured ATP synthesis rates in the most HTS (*Bellapiscus medius*) and the HSS (*Forsterygion varium*) at pH 7.25 and 6.65. Mitochondria from HTS brain did have greater H^+^ buffering capacities than HSS *mt* (∼10 mU pH.mg_protein_^-1^). HTS *mt* swelled by 40% when exposed to a decrease of 1.5 pH units, and JO_2_ was depressed by up to 15% in HTS. However, HTS were able to maintain ΔΨm near -120 mV. Estimates of work, in terms of charges moved across the *mt* inner-membrane, suggested that with acidosis, HTS *mt* may in part harness extra-*mt* H^+^ to maintain ΔΨm, and could therefore support ATP production. This was confirmed with elevated ATP synthesis rates and enhanced P:O ratios at pH 6.65 relative to pH 7.25. In contrast, *mt* volumes and ΔΨm decreased downward pH 6.9 in HSS *mt* and paradoxically, JO_2_ increased (∼25%) but ATP synthesis and P:O ratios were depressed at pH 6.65. This indicates a loss of coupling in the HSS with acidosis. Overall, the *mt* of these intertidal fish have adaptations that enhance ATP synthesis efficiency under acidic conditions such as those that occur in hypoxic or reoxygenated brain.

## Introduction

In hypoxic or anoxic conditions, O_2_ becomes limiting and ATP production via mitochondrial (*mt*) oxidative phosphorylation (OXPHOS) is compromised. To support ATP requirements, vertebrate cells increase anaerobic metabolism activities, which is ∼15-fold less efficient than the OXPHOS. If hypoxia is sustained, glycolysis may become substrate limited, and diminishing ATP production mediates rapid depletion of ATP stores (Pamenter, 2014). ATP hydrolysis mediates proton (H^+^) release (Wilson, 1988) alongside the accumulation of metabolic end-products (Azarias et al., 2011), which contributes to metabolic acidosis (Robergs et al., 2004). Although lactate is possibly oxidized by neurons (Quistorff et al., 2008; Gallagher et al., 2009; Barros, 2013; Riske et al., 2017) this requires oxygen, and lactate accumulation contributes to intracellular acidosis (reviewed in Kraut and Madias, 2014). In the ischemic brain, up to 60% of glucose can be metabolized to lactate (Teixeira et al., 2008; Dienel, 2012), which the accumulation of has been shown to associate with hypercarbia and acidosis (Rehncrona, 1985a; Katsura et al., 1992b).

Acidosis alters *mt* respiration in ischemic mammalian brain (Hillered et al., 1984), enhances brain lipid peroxidation *in vitro* (Siesjo et al., 1985) and denatures proteins (Kraig and Wagner, 1987). Low pH (<6.8) also inhibits the hydrolytic role of F0F1-ATP synthase in isolated myelin vesicles (Ravera et al., 2009), and acidosis generally promotes irreversible cellular damage (Rehncrona, 1985a,b; Rehncrona and Kagstrom, 1983). In most vertebrates, acidosis occurs rapidly and compromises brain function within minutes of anoxia (Katsura et al., 1991). Hypoxia tolerant species (HTS) however, routinely survive hypoxic or anoxic environments for several hours to months, which make these animals useful model systems to explore adaptations against hypoxic damage.

Adult vertebrates such as the carp (*Carassius carassius*), its cousin goldfish (*C. auratus*) and the freshwater turtle (*Chrysemys picta*) have strategies that decrease lactate-mediated acidosis (Jackson, 2004; Vornanen et al., 2009). Among mammalian hibernators, the artic ground squirrel (*Spermophilus parryii*) suffers little damage from ischemia while torpid at body temperatures as low as -3°C (Barnes, 1989; Ma et al., 2005). Independent of hibernation cycle, normothermic brain slices of the ground squirrel tolerate O_2_, ATP and glucose deprivation (Bhowmick et al., 2017). However, determining how such physiological adaptations to hypoxia evolve is harder to explore. Comparative approaches using multiple phylogenetically related species can provide insights (Pagel, 1997) in particular if species range in their tolerances to hypoxia.

The New Zealand triplefin fish group (Family *Tripterygiidae*) consists of 26 endemic species, most of which occupy stable normoxic habitats and are considered to be HSS. However, three HTS species are apparent, and these have evolved to inhabit the intertidal zone that can become hypoxic at low tide (Hickey and Clements, 2005; Hilton et al., 2008; Hilton, 2010; Richards, 2011). In our previous work, the hypoxia-tolerance of intertidal triplefins, such as *Bellapiscis medius* show significantly greater tolerance to hypoxia with a lower critical O_2_ pressure (P_crit_), while subtidal species such as *Forsterygion varium* had significantly higher P_crit_ (Hilton et al., 2010). Moreover, the intertidal triplefin species have elevated anaerobic enzymes and pH buffering capacities in skeletal muscle (Hickey and Clements, 2003), which likely extend energy production and prevent acidic damage. In addition, there appears to have been selective pressures on the *mt* genomes of rock-pool species relative to subtidal species (Hickey et al., 2009), suggesting aerobic metabolic pathways may have been influenced by the stress of life in the intertidal zone.

The close genetic background within this group (Hickey and Clements, 2005) make these fish a natural model to understand adaptations, such as those to survive hypoxic environments. Therefore, we selected four triplefin species with various degrees of hypoxia tolerance. *B. medius* was our exclusive HTS, as this species occupies high rock pools. The more generalist species *F. lapillum* and *F. capito* yet have a marginally lower tolerance to hypoxia and served as intermediates between the HTS and the HSS *F. varium* occupying stable subtidal waters do not typically encounter hypoxia. We hypothesized that intertidal triplefins will show *mt* adaptations commensurate with physiological stressors associated with hypoxia. As *mt* respiration (JO_2_) regulates the *mt* membrane potential (ΔΨm) and maintains a pH gradient (Mitchell, 2011), we tested the influence of lactate mediated acidosis on brain *mt* of triplefin fish, and predicted that *mt* of HTS would maintain function at lower pH compared to HSS.

## Materials and Methods

### Animal Sampling and Housing

Adult specimens of four triplefin species (5–10 cm) were collected from different sites around the greater Auckland region using hand nets and/or minnow traps. Adult *B. medius* were caught from high rock-pools at low tide, *F. lapillum* and *F. capito* from rock-pools and off piers, and *F. varium* at 5–10 m depth. Individuals were maintained in 30 L tanks (20 fish per tank) in recirculating aerated seawater and were fed with a standard mixture of shrimps and green-lipped mussels every 2 days for a 2 weeks acclimation period prior to experiments procedure at 20 ± 1°C. All capture, housing and experimental procedures were performed with under the approval from the University of Auckland Ethic Committee (Approval R001551).

### Brain Preparation and Tissue Permeabilization

Fish were euthanized by section of the spinal cord at the skull. The brain was immediately removed and placed in a modified ice-cold biopsy buffer containing (in mM from hereon unless stated) 2.77 CaK_2_EGTA, 7.23 K_2_EGTA, 5.77 Na_2_ATP, 6.56 MgCl_2_.6H_2_O, 20 taurine, 15 Na_2_-phosphocreatine, 20 imidazole, 0.5 DTT, 50 KMES, 50 sucrose, pH 7.1 at 30°C (Gnaiger et al., 2000). Cellular permeabilization was undertaken by the addition of 50 μg ml^-1^ freshly prepared saponin and 30 min of gentle agitation within cell culture plastic plates held on ice. The permeabilized tissue was then removed and washed three times for 10 min ice-cold modified MiR05 respiration medium (Kuznetsov et al., 2000) containing 0.5 EGTA, 3 MgCl_2_.6H_2_O, 60 K-lactobionate, 20 taurine, 10 KH_2_PO_4_, 2.5 HEPES, 30 MES, 160 sucrose, 1 g.l^-1^ BSA, pH 7.1 at 30°C. Brain tissues were then split longitudinally into two halves, blotted dry on filter paper, and weighed before loading into respirometers.

### Mitochondrial Isolation From Minimal Fish Brain Tissues

A miniaturized *mt* isolation was required to assess the *mt* swelling. Triplefin brain masses varied from only ∼8–30 mg of tissue, hence the whole triplefin brain was required for *mt* isolation. The brain was first removed from the skull and gently homogenized in 1 ml cold MiR05 by expulsion and suction through a modified 1 ml syringe with decreasing gauge needles (16–25 gauge). Mitochondrial integrities were better preserved with this method compared to other standard homogenization methods and the small sample was also better retained (personal observation). The homogenate (600 μl) was centrifuged at 300 × *g* for 5 min at 4°C, the supernatant, which contained suspended *mt* was collected and spun at 11,000 × *g* for 10 min. The supernatant and the white lipid ring surrounding the brown *mt* rich pellet was discarded prior to the addition of 500 μl cold MiR05. The last step was repeated twice and isolated *mt* were then re-suspended in 50 μl ice-cold MiR05 and were held on ice for 1 h to permit recovery before respirometry assays. Post respirometry assays, the medium containing the *mt* was removed from the chambers and stored at -80°C for the determination of protein concentration. Prior to the protein assay, samples were slowly defrosted at 4°C and the protein concentration was determined with the Pierce^TM^ BCA Protein Assay Kit as specified by the manufacturer, against a BSA standard and modified MiR05 control.

### Acidification Protocol Optimization

**FIGURE 1:**
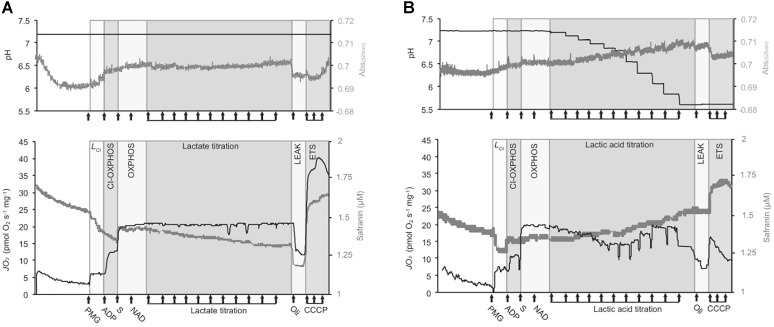
Representative traces of the respirometry protocol. Experiments were conducted with Oroboros^TM^ O2ks on isolated *mt* and permeabilized brain from triplefin fish. The medium pH (top panels, black traces), Vol_mt_ (top panels, gray traces), *mt* respiration rates (“JO_2,_” lower panel, black traces) and *mt* membrane potential (ΔΨm, lower panels, gray traces) are presented in two different graphs. However, a maximum of three parameters (JO_2_, medium pH and ΔΨm assessed on permeabilized brain; or JO_2_, medium pH and Vol_mt_ assessed on isolated *mt*) was assessed in a single experiment. Permeabilized brain or isolated *mt* from triplefin fish brain were introduced in the O2k chamber and left to recover and signals to stabilize for 20 min. The LEAK state supported by CI (*L*_CI_) was mediated by the addition of CI-linked substrates (PMG for pyruvate, malate, and glutamate) at saturating concentrations. The addition of ADP then activates CI-mediated OXPHOS and the addition of saturated succinate (S) allowed the measurement of full OXPHOS capacities with the contribution of both CI and CII. Then, NAD^+^ (75 μM) was added to stimulate extra-*mt* lactate oxidation into pyruvate. Buffered lactate (control samples) or unbuffered lactic acid was then titrated to 30 mM (**A** and **B**, respectively). To test for *mt* coupling, the ATP_F0-F1_ inhibitor oligomycin (Oli) was added to determine the LEAK state (affected or not by pH) followed by the uncoupler CCCP, titrated to define the maximum ETS capacity. The ΔΨm was calculated from the fluorescence signal recorded from a calibrated-safranin concentration (up to 2 μM). An increase in safranin concentration corresponds to a decrease in ΔΨm. Vol_mt_ was estimated with the absorbance at 525 nm. All experiments were performed at 20°C with sufficient O_2_ (between 100 and 260 μM dissolved O_2_).

To explore the influence of pH on *mt* function we chose to titrate lactic acid into a modified buffer to induce acidosis. Although acidosis results from ATP hydrolysis, other glycolytic and TCA intermediates and CO_2_ (Roos and Boron, 1981), and whether lactate ionizes to an acid *in vivo* is contentious (Robergs et al., 2004), ULac provides an organic acid without inorganic ions [such as Cl^-^ if HCl were to be used (Selivanov et al., 2008)]. Given the high buffering capacity of typical respiration media (MiR05) (Gnaiger et al., 2000), we decreased the pH buffering to mimic the pH changes expected in hypoxic brain, which may decrease to an extracellular pH of 6.3 in ischemic brain of non-hyperglycemic vertebrate brain (Katsura et al., 1991, 1992a; Kraut and Madias, 2014) and of ∼6 *in vivo*, at least in hyperglycemic mammals (Katsura et al., 1991, 1992a). Assuming parallel changes in pH, these changes should equate to intracellular pH ∼5.6–5.5. Therefore, we decreased the HEPES concentration to 2.5 mM and used 30 mM MES to buffer at low pH. With this buffer system, ULac/or BLac (pH ∼7 adjusted with KOH) was titrated to cover physiological concentrations (Jokivarsi et al., 2007; Witt et al., 2017), and the pH changes mediated by ULac covered those that occur within vertebrate ischemic brain with a decrease 0.048 pH units per mM ULac. The media (or extra-*mt* pH) was recorded simultaneously with respiration and *mt* membrane potential (ΔΨm) using a solid state ISFET electrode (IQ Scientific Instruments) connected to the pX port of the O2k and calibrated using a three-point calibration (pH 4, 7, and 10) prior to experiments and allowed a ±0.001 pH U sensitivity. Measurements were performed on permeabilized brain and isolated *mt*, and pH buffering capacities were calculated relative to no-sample controls. As lactate is oxidized by LDH to produce NADH^+^ and pyruvate, which is further oxidized by *mt* for oxidative phosphorylation (OXPHOS) (Halestrap, 1975), it likely alters JO_2_, ΔΨm, and pH in the presence of other respiratory substrates. Therefore, the influence of pH changes mediated by ULac was made relative to BLac controls. A representative trace of the protocol is displayed in Figure 1.

### Respirometry

Between 5–12 mg of tissue was placed in respirometer chambers (Oroboros Instrument, Innsbruck, Austria) containing 2 ml (or 3.4 ml when extra-*mt* pH measured to accommodate the electrode) oxygen saturated modified-MiR05 (O_2_ concentration = 290 μM at 20°C and 101.5 kPa barometric pressure). Two substrate uncoupler inhibitor titration protocols were performed. The first assay informed on the lactate-mediated respiration and consisted in the sequential addition of NAD^+^ (75 μM) and BLac (30 mM, pH 7.25) to initiate a leak state measurement (LEAK), followed by the addition of ADP (700 μM) to initiate OXPHOS. The second assay was designed to assess the effect of pH on *mt* at OXPHOS state and consisted of the subsequent addition of the NADH_2_-generating substrates pyruvate (10 mM) malate (5 mM) and glutamate (10 mM) to initiate LEAK. OXPHOS supported by CI was then commenced by the addition of ADP (700 μM). The subsequent addition of 10 mM succinate activated parallel inputs from CI and CII to OXPHOS. NAD^+^ (75 μM) was added to the media to avoid cytosolic limitations (i.e., LDH and malate aspartate shuttle, discussed in Kane, 2014). BLac or ULac was then titrated to a 30 mM final concentration. Then, the F0F1-ATP synthase inhibitor oligomycin (2.5 μl) was added to place *mt* into artificial LEAK state. Subsequently, respiration was uncoupled from OXPHOS using three injections of the protonophore carbonyl cyanide m-chlorophenyl hydrazone (CCCP, 0.5 μM each) to determine the maximal ETS capacity. O_2_ concentration was maintained above 100 μM to avoid diffusion limitation. The protocol was applied on both isolated *mt* and permeabilized brain, however, only respiration and ΔΨm data from permeabilized brains are presented in the main manuscript (please refer to Supplementary Figures for isolated *mt* respiration data). The differences due to changes in pH within the same permeabilized brain in term of JO_2_, ΔΨm and *mt* matrix volume (Vol_mt_) were determined by referencing experiments with ULac relative to those with BLac (detailed comparison in Supplementary Figure S1).

### Mitochondrial Volume (Vol_mt_) Dynamics

Mitochondria are dynamic organelles and shrink or swell when exposed to variable conditions (Cereghetti and Scorrano, 2006; Casteilla et al., 2011; Friedman and Nunnari, 2014). Changes in *mt* volume (shrinkage or swelling) of isolated *mt* were measured by following changes in absorption at 525 nm, which changes proportionally to the volume of the *mt* matrix (Beavis et al., 1985; Garlid and Beavis, 1985; Das et al., 2003). Fluorescence sensors (green LED, 525 nm I_50_ ± 25 nm) were used without an emission filter, to follow the light absorption within the O2k. The voltages were recorded simultaneously with JO_2_, ΔΨm, and pH and light absorbance was calculated with:

$$A_{\lambda }=-\log_{10}\frac{\left(I-D\right)}{\left(R-D\right)}/W$$

Where A_λ_ corresponds to the total absorbance, *I* to the sample intensity, *D* to the dark intensity, *R* to the reference intensity and *W* to the amount of protein in mg. Changes in absorbance ΔA_λ_ were normalized by Aλ in the OXPHOS state to account for the effect of pH on phosphorylating *mt* only and where *mt* shrinkage and swelling occurs when ΔA_λ_ < 1 and ΔA_λ_ > 1, respectively.

### ΔΨm Measurement and Calculation

Safranin-O was used to estimate ΔΨm, simultaneously with JO_2_ and pH measurements on permeabilized brain and isolated *mt*. Safranin is a cationic dye that undergoes a fluorescent quench upon its movement from the intermembrane space (IMS) into anionic sites within the *mt* matrix (Akerman and Wikstrom, 1976; Zanotti and Azzone, 1980). A near-linear correlation between the safranin spectral shift and mitochondrial energized state permits estimates of ΔΨm. As recommended, [Safr]_final_ of 2 μM was chosen for this study (Krumschnabel et al., 2014) and the fluorescence signal (Ex/Em 465/587 nm) was calibrated using a four-step titration (0.5–1–1.5–2 μM) into the O2k chamber prior to experiments. Although the signal in control experiments (no sample) was unchanged by the addition of different substrates-inhibitors, nor by changes in pH, quenching occurred over time (3 nM min^-1^) and was accounted for in ΔΨm calculations. ΔΨm was calculated from the recorded concentration of safranin “[Safr]” as per previous work (Pham et al., 2014), where:

$$\Delta \psi m=2.3026\times \frac{RT}{zF}\times Log10\left(\frac{[Safr]out}{[Safr]in}\right)$$

Where *R* is the gas constant, *T* is the temperature in Kelvin, *z* the valence state of the ion (+1) and *F* the Faraday constant. While the safranin concentration outside the *mt* “[Safr]_out_“corresponds to the calibrated fluorescent signal directly obtained from DatLab7, the safranin concentration in the *mt* matrix “[Safr]_in_“is dependent on the Vol_mt_, back-calculated from OXPHOS state, which was assumed to be at -120 mV (Huttemann et al., 2008; Perry et al., 2011). As *mt* shape is dynamic (Fujii et al., 2004; Casteilla et al., 2011), the Vol_mt_ was readjusted at each state following the estimation and the integration of the *mt* swelling or shrinkage (ΔA_λ_):

$${Vol}_{mt}=(\mu l{mg}^{-1})=\frac{Safr\times 10^{-\frac{120}{Cst}}}{[Safr]out\times W}10^{6}\times \Delta A_{\lambda }\,$$

Where *Cst* corresponds to $2.303\times \frac{RT}{zF}$ (58.17 mV) under our conditions, *Safr* the amount of safranin in *mt* in μmol, [Safr]_out_ the recorded safranin concentration in μM, *W* the amount of tissue in mg and ΔA_λ_ the change in Vol_mt_ (described above).

### Estimation of Mitochondrial Work to Maintain ΔΨm With Additional External Charges

We further estimated the energy required by *mt* to maintain ΔΨm as this summarizes the combined effects of JO_2_, ΔΨm, and acidosis. The total energy of a closed system can be determined by:

J=C.V⁢

Where *J* is the derived unit of energy transferred to an amount of work in Joules, *C* is electric charge in Coulombs and *V* is the electric potential in Volts. A Joule is the work required to move an electric charge of 1C against an electric potential of 1 V. In this present study, we treat *mt* as a closed system and the respiration rate a measure of the work performed by complexes to transfer H^+^ across the mitochondrial inner membrane (MIM) for each O_2_ reduced against ΔΨm. Proton pumping varies from 12 H^+^ to 20 H^+^ per O_2_ consumed with full support from CII or CI (respectively). Therefore, the relative contribution of CI to OXPHOS was calculated and total proton pumping per O_2_ determined:

$$nH^{+}=12+8*\frac{{OXPHOS}_{\left(pyruvate+malate+glutamate\right)}}{{OXPHOS}_{\left(pyruvate+malate+glutamate+succinate\right)}}$$

The proton flux was then calculated:

$$J\left(nH^{+}\right)={JO}_{2}*nH^{+}$$

The equation (4) was transposed:

$$J_{mt}=e*J\left(nH^{+}\right)*\Delta \Psi m$$

Where *e* equals the elementary charge constant corresponding for H^+^ to 96.525 kC mol^-1^, ΔΨm the *mt* membrane potential expressed in mV and *J*(*n*H^+^) the rate of protons passed through the MIM in pmol H^+^ s^-1^ mg^-1^. While other ions contribute to ΔΨm *in vivo*, only K^+^ and Cl^-^ ions in our media impact ΔΨm, and these remain constant relative to the changes in H^+^.

To better relate mitochondrial work to equation 4, we thought to calculate the amount of charge (in Coulomb, “C_add_”) mediated by the decrease in pH (i.e., additional protons), where:

$$C_{add}=10^{-pH}*V_{chamber}*N_{A}*C_{H}\,$$

Where pH is the recorded pH in the chamber, V_chamber_ is the volume of the respirometry chamber (2 ml), N_A_ is the Avogadro constant (6.02.10^23^) and C_H_ the charge carried by a proton (1.602 × 10^-19^ C).

At stable ΔΨm, charges moved from the *mt* matrix to the inter-membrane space equates those moved back to the matrix. To better represent the effect C_add_ at a given pH onto charges flux within *mt*, *J*_mt_ was normalized by C_add_ at corresponding pH.

### ATP Production

ATP production was assessed based on previous work (Chinopoulos et al., 2009; Pham et al., 2014; Masson et al., 2017) in permeabilized brain of the most HTS *B. medius* and the HSS *F. varium*. Due to the variable properties of the fluorescent dye Magnesium Green^TM^ (Thermo Fisher Scientific, United States) to acidosis, we performed an experiment to assess ATP production at pH 7.25 and pH 6.65, which appears to be the pH at which *mt* function is preserved in HTS but altered in the HSS (Figure 4). In separated experiments, O2k chambers were filled with modified MiR05 and mitochondrial substrates (pyruvate, malate, glutamate, and succinate) at concentrations described above. This was supplemented with 10 mM BLac or ULac to set the medium pH at 7.25 or 6.65, respectively. Magnesium Green^TM^ (5 μM) was then added and MgCl_2_ was titrated to calibrate the Mg^2+^_free_ in the chamber. The equivalent of one permeabilized brain hemisphere was then added to the chamber and let to recover for around 15 min. Then, ADP was titrated (3 × 0.5 μM) to calibrate the Mg^2+^_free_ signal to [ADP]. After 15 min, antimycin A (2.5 μM) was added to block proton pumping by the ETS. Once the JO_2_ signal was stable (>20 min), oligomycin (10 nM) was added to block mitochondrial ATP synthesis and measure ATP hydrolysis. Data was exported into Excel and the rate of ATP was calculated as:

$$JATP=\frac{{\left[ADP\right]}_{t}-{\left[ADP\right]}_{t-1}}{t-t_{-1}}*Cst$$

Where [ADP]_t_ corresponds to the concentration of ADP at the end of a *mt* state and [ADP]_t-1_ the ADP concentration at the start of the *mt* state and t and t-1 the time at which corresponding [ADP] were taken. Cst corresponds to the relative difference between K_DADP_ and K_DATP_ extracted from Chinopoulos et al. (2009). Then ATP consumption rate determined after the addition of oligomycin (JATP < 0), was subtracted to the other state to determine the net ATP production rate (JATP > 0) in pmol s^-1^ mg^-1^. The PO ratio was calculated as:

$$PO=\frac{JATP}{\frac{{JO}_{2}}{2}}$$

### Data and Statistical Analysis

Respirometry and simultaneous spectrometry data were extracted from DatLab 7.0 software. All data were copied and processed in Excel © 2016. GraphPad Prism v7 was used to perform two-way ANOVA to test for the effect of pH between species and the *mt* parameters (JO_2_, ΔΨm, Vol_mt_, and ATP) within *mt* states. Two-way ANOVA repeated-measures were performed to analyze the effect of BLac, ULac, and associated pH titrations on the *mt* parameters between the species of fish. *Post hoc* tests using Turkey’s correction were used for pairwise comparisons and a *P*-value of 0.05 was chosen to represent statistical difference.

## Results

### Mitochondrial pH Buffering Capacities

Permeabilized brain of *B. medius* had a 4.5–6% (∼0.05 pH-unit mg^-1^) greater buffering capacities than the HSS *F. varium* (*P* = 0.006; Table 1). Brain tissue pH buffering capacities of *F. lapillum* were similar to those of the more HTS *B. medius*, while *mt* of *F. capito* had similar buffering to *F. varium*. In isolated *mt*, however, only *F. varium* differed, with a lower pH buffering capacity (*P* < 0.04), indicating most, but not all, of the buffering results from non-*mt* components. Estimated contributions of *mt* to brain buffering capacities approach ∼20% in HTS and 10% in HSS brain.

**Table 1:** pH buffering capacities, mitochondrial volume and efficiency of permeabilized tissue and isolated *mt* from triplefin brain.

	pH buffering		Vol_mt_ (μl mg_permeabilized brain_^-1^)	RCR	
			
	Permeabilized (pH-unit mg_brain_^-1^)	Isolated *mt ^#^* (pH-unit mg_protein_^-1^)	OXPHOS (at -120 mV)	Permeabilized	Isolated *mt*
*B. medius*	0.940 ± 0.007 *^cv^*	0.193 ± 0.059 *^v^*	2.06 ± 0.24 *^v^*	2.66 ± 0.25	6.64 ± 1.64 ^#^
*F. lapillum*	0.929 ± 0.011 *^v^*	0.169 ± 0.072 *^v^*	2.09 ± 0.25 *^v^*	3.38 ± 0.51	3.17 ± 1.29
*F. capito*	0.892 ± 0.005 *^m^*	0.177 ± 0.028 *^v^*	2.14 ± 0.18 *^v^*	2.42 ± 0.10	1.79 ± 0.10
*F. varium*	0.889 ± 0.008 *^m^*	0.089 ± 0.062 *^mlc^*	1.53 ± 0.21 *^mlc^*	1.94 ± 0.15	6.74 ± 0.73 ^#^


*Buffering capacities were calculated relative to controls (no sample) in permeabilized brain and isolated *mt* at OXPHOS state exposed to graded acidosis, mediated by acid lactic titration (0–30 mM, 7.24–5.73 pH equivalent). Results expressed as mean (*n* = 5) ± SEM. The *mt* volume (Vol_*mt*_) was back-calculated from the membrane potential signal as per described in the “Materials and Methods” section, with the assumption that in phosphorylating *mt*, the membrane potential equates -120 mV. Data expressed as mean of *n* = 7 ± SEM. Respiratory control ratio (RCR) in both permeabilized and isolated *mt* and relates to coupling efficiencies. Data expressed as mean of 7 ± SEM. Significant difference at *P* < 0.05 from designated species between *B. medius*, *F. lapillum*, *F. capito* or *F. varium* indicated as *m*, *l*, *c* and *v*, respectively. The difference between sample preparation states was chosen at *P* < 0.05 and indicated as # (all tested with two-way ANOVA with Turkey correction).*

### Overall Mitochondrial Oxygen Flux and pH Effects on the Mitochondrial Function

**FIGURE 2:**
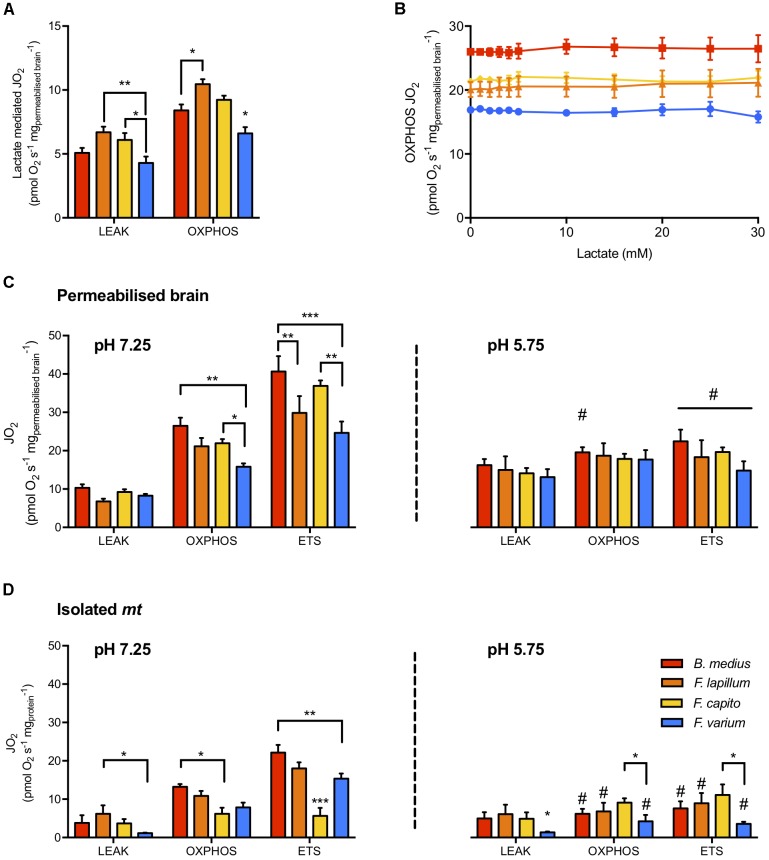
Mitochondrial respiration rates and the effect of lactate and pH on mitochondrial states. **(A)** The capacity of lactate (30 mM) to fuel LEAK and OXPHOS respiration in the absence of other mitochondrial substrates. **(B)** Titration of buffered lactate (pH 7.25) on mitochondria in OXPHOS state, in the presence of saturating CI and CII substrates (pyruvate, malate, glutamate and succinate), ADP and NAD^+^. In permeabilized brain **(C)** and isolated mitochondria **(D)**, the respiration attributed to OXPHOS was determined with 30 mM buffered lactate. The portion of respiration attributed to proton leak (LEAK) was measured with the further addition of oligomycin and the maximum capacity of the electron transport system (ETS) was measured as the maximum respiration uncoupled from OXPHOS, reached with the titration of the uncoupler CCCP. Experiments were conducted at physiological pH (left panel) and acidic conditions (pH 5.75, right panel) mediated with 30 mM lactic acid instead of buffered lactate. Hypoxia tolerant species are presented in warm colors while the hypoxia sensitive species is presented in blue. Results presented as mean ± SEM of *n* = 7 individuals in **(A,C)** and *n* = 5 in **(B)**. Significance difference between species at *P* < 0.05; 0.01 or 0.001 indicated as ^∗^, ^∗∗^, ^∗∗∗^, respectively, and between pHs (within a mitochondrial state) chosen at *P* < 0.05 and indicated as # (two-way ANOVA and *post hoc* test with Turkey correction).

In phosphorylating *mt* (presence of sufficient *mt* substrates and ADP), BLac addition did not alter OXPHOS JO_2_ in any species (*F*_10,60_ = 0.381, *P* = 0.95; Figure 2A). However, in the absence of other *mt* substrates, lactate mediated JO_2_ in the HTS *mt* > 22% more than in *F. varium mt* (*P* < 0.03; Figure 2B) and *F. lapillum* JO_2_ was also 30% greater than *B. medius* (*P* < 0.01).

The JO_2_ differed among species for permeabilized brain and isolated *mt* held at a physiological pH (species effect *F*_3,48_ = 15.8 and *F*_3,72_ = 10.5, respectively, *P* < 0.01; Figure 2). While the LEAK JO_2_ in permeabilized brain was similar among species (Figure 2C), OXPHOS and ETS JO_2_ were greater in *B. medius* and *F. capito* relative to *F. varium* (*P* < 0.05). No difference was observed between *F. lapillum* and *F. capito*, which JO_2_ at both OXPHOS and ETS states, sat between JO_2_ of the two other species. All species had greater ETS fluxes than OXPHOS fluxes (*P* < 0.02) indicating some limitation of the OXPHOS system relative to the ETS, and this was further observed in isolated *mt* (Figure 2D).

In the presence of 30 mM ULac, which mediated a decrease of pH to 5.75, JO_2_ was similar between species and across all states in permeabilized brain (*P* = 0.26; Figure 2C). However, while LEAK was increased by ∼50% with ULac relative to BLac (*F*_1,6_ = 47.4, *P* < 0.001), ETS was significantly decreased by ∼50% (*F*_1,6_ = 139, *P* < 0.001). In OXPHOS, there was interaction between species and pH (*F*_3,18_ = 4.35, *P* = 0.02), mediated by a significant decrease in JO_2_ in *B. medius* (*P* = 0.02) only. In isolated *mt* (Figure 2D), acidosis did not affect *F. capito*, but decreased OXPHOS and ETS by >50% in all other species (*P* < 0.05). OXPHOS was 50% lower and ETS was 65% lower in *F. capito* than in *F. varium mt* (*P* = 0.05 and 0.009, respectively).

**FIGURE 3:**
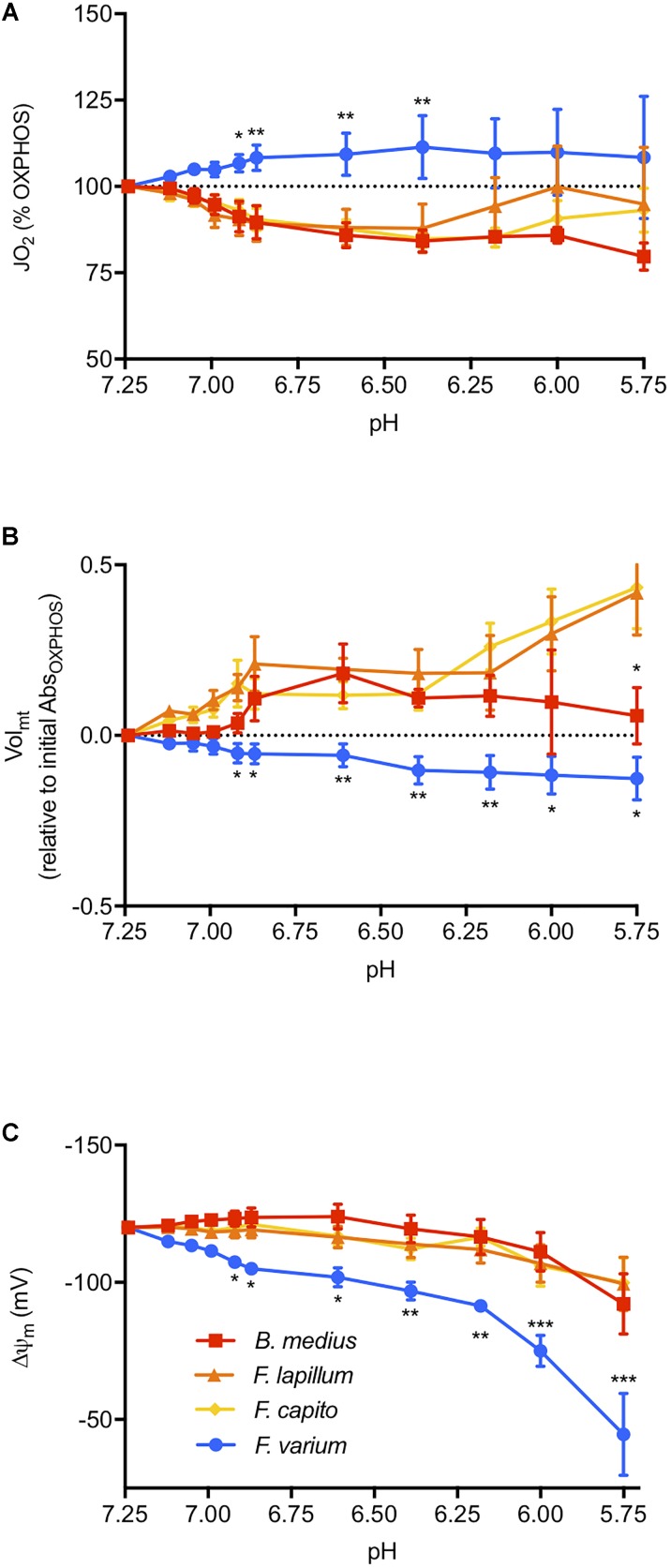
The effect of pH on the mitochondrial function. Respiration rate (JO_2_, **A**), mitochondrial volume (Vol_mt_, **B**), and membrane potential (ΔΨm, **C**) were measured at in the presence of saturating mitochondrial (*mt*) substrates and ADP in chambers of oximeters at 20°C (see the “Materials and Methods” section for more details). Buffered lactate (controls, constant pH) or unbuffered lactic acid (to decrease the medium pH) was then titrated in parallel respirometry chambers up to 30 mM and the lactate acid results were subtracted from the lactate results to account for the specific effects of pH on the mitochondrial parameters. ΔΨm was calculated from the fluorescence of safranin-O and with the consideration of the change in Vol_mt_, calculated with the change in absorbance at 525 nm. Overall, acidosis mediated a contrasted response between the hypoxia sensitive species (blue) and the hypoxia-tolerant species (warm colors), with an inhibition of JO_2_ and greater maintenance of ΔΨm induced with mitochondrial swelling in HTS. Results presented as mean (*n* = 7) ± SEM. For clarity, only the difference between *F. varium* and the other species is displayed as ^∗^, ^∗∗^, and ^∗∗∗^ for *P* < 0.05; 0.01 or 0.001 (main effect of pH and species tested with two-way ANOVA repeated measures).

We then assessed the effect of graded acidosis on OXPHOS, which overall, mediated a contrasting response between the HTS and the HSS (main effect of species *F*_3,18_ = 4.42, *P* = 0.02; Figure 3). First, JO_2_ was gradually decreased in HTS until around pH 6.4 to ∼18% relative to OXPHOS_initial_ (at pH 7.25, Figure 3A). Below pH 6.4, the response was more variable in the *Forsterygion* genus, whereas JO_2_ was more stable in *B. medius*. In *F. varium*, however, JO_2_ increased by 12% and was more variable below pH 6.9. JO_2_ was significantly different between *B. medius* and *F. varium* below pH 7 (*P* < 0.01).

### Membrane Potential and Changes in Mitochondrial Volume (Vol_mt_)

The Vol_mt_ in HTS was 30% higher than *F. varium* in OXPHOS (*P* < 0.04, Table 1). Below pH 6.9, acidosis mediated swelling in HTS *mt*, and decreased Vol_mt_ in *F. varium mt* (interaction between species and pH of *F*_30,120_ = 3.59, *P* < 0.001; Figure 3B). While the Vol_mt_ increased to around 50% in *F. capito* and *F. lapillum*, it decreased by 12% in *F. varium*, relative to Vol_mt_ at pH 7.25 (*P* < 0.04). Differences between *B. medius* and *F. varium* were significant below pH 6.6 (*P* < 0.03).

Using estimates of Vol_mt_ dynamics with pH changes, we incorporated Vol_mt_ into the Nernst equation to better derive the ΔΨm relative to pH (Supplementary Figure S2). Two-way ANOVA revealed an interaction between ΔΨm, species and pH (*F*_30,180_ = 3.71, *P* < 0.001, Figure 3C). While the main effect of pH was significant for all species (*F*_3,18_ = 9.39, *P* = 0.001), only ΔΨm at pH 5.75 (around -100 mV) differed from its original value at pH 7.25 (-120 mV) in the HTS. In contrast, ΔΨm in *F. varium mt* gradually decreased down to around -50 mV at pH 5.75 and was significantly lower than ΔΨm *in B. medius mt* (*P* < 0.05 from pH 7). For comparison, a ΔΨm of -110 mV was reached at pH 7 in *F. varium* and 6.12–6 in the HTS.

### Energy Attributed to Sustaining ΔΨm

**FIGURE 4:**
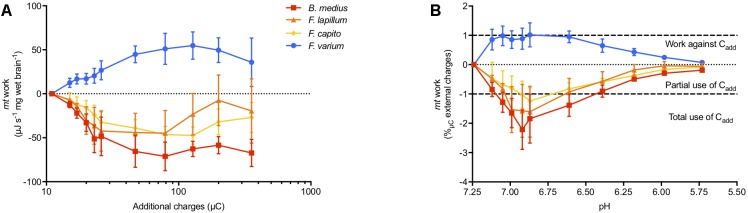
Mitochondrial work exposed to external charges loads mediated by acidification. Work is defined here by the protons/charges transferred against the ΔΨm. At OXPHOS state, the mitochondrial respiration rate was used as proxy for the proton pumping, calculated with the relative contribution of the mitochondrial complexes to respiration (between 12 and 20 H^+^ pumped per O_2_ consumed). ΔΨm was accurately calculated accounting for mitochondrial volume dynamics and the extra-*mt* charges were calculated from the measurement of medium pH, adjusted with lactic acid titration on permeabilized brain from triplefin fishes. **(A)** Mitochondrial work was then calculated and expressed as the mass specific energy (μJ s^-1^ mg^-1^) used to transfer protons and plotted against the additional charges (μC) mediated by acidosis (additional H^+^). **(B)** The relative work exercised by *mt* was then normalized against additional positive charges (C_add_) and plotted against the medium pH. Positive values indicate the use of internal energy derived from the ETS to develop the ΔΨm against C_add_, whereas negative values represent the portion of C_add_ that contributes to ΔΨm maintenance. While the hypoxia sensitive *F. varium* exerts positive work against acidosis, the other species seem to take advantage of external charges to maintain ΔΨm. Results of *n* = 7 individuals per species, expressed as mean ± SEM.

Combined JO_2_, ΔΨm, and extra-*mt* pH data allowed an estimate of the work by *mt* to develop ΔΨm in OXPHOS state in regard to additional positive charges mediated by addition of acid (“C_add,_” additional H^+^) (Figure 4). Brain *mt* from *F. varium* showed graded increase in work with C_add_, up to ∼50 μJ s^-1^ mg^-1^ at 100 μC charges (pH 6.6 equivalent, Figure 4A). In contrast, the remaining species showed a graded decrease in work down to -50 μJ s^-1^ mg^-1^ at ∼25 μC (pH 7). We then normalized the *mt* work by C_add_, which represents the use of C_add_ against or used to develop ΔΨm (Figure 4B). While internal work was maximized in *F. varium* with the first decrease in pH (from 7.25 to 7.12), C_add_ were fully used by *B. medius* to develop ΔΨm (*P* = 0.02). Between pH 7.12–6.4, internal work was further decreased in *B. medius* (*P* < 0.04), although increased for *F. varium* (*P* < 0.04). C_add_ was appeared to be utilized by *F. lapillum* and *F. capito mt* between pH 7.05–6.6 and pH 6.9–6.6, respectively (*P* < 0.05). Progressively, *mt* work (positive in *F. varium* and negative in HTS) returned to near zero values at approximately pH 6.

### ATP Production

**FIGURE 5:**
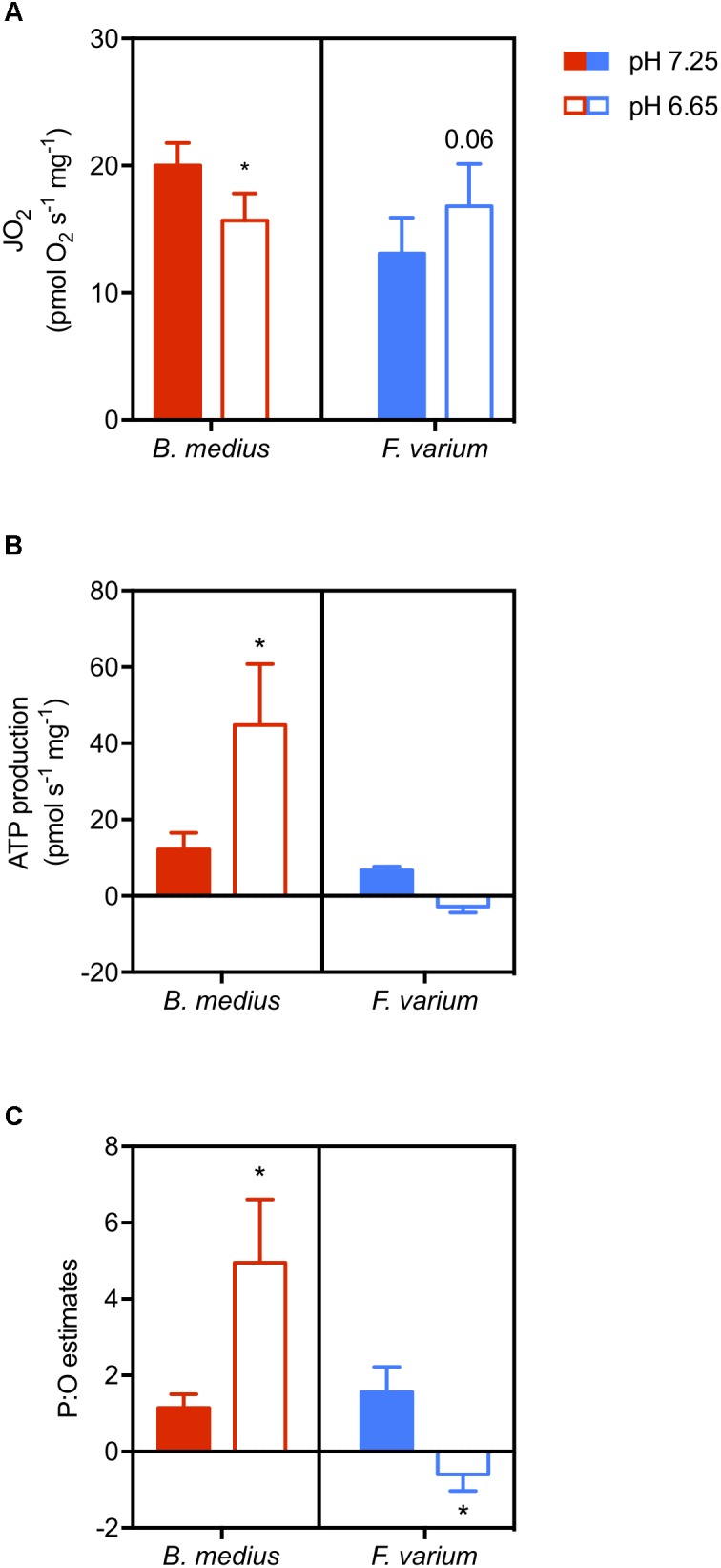
Acidosis enhances ATP production in brain mitochondria of the intertidal species. Permeabilized brain hemispheres were placed in respiratory chamber with mitochondrial substrates (described in the “Materials and Methods” section) and 10 mM buffered lactate or unbuffered lactic acid to set the respiratory medium pH at 7.25 or 6.65, respectively. Mitochondrial respiration (“JO_2,_” **A**) was measured simultaneously with ATP production **(B)** using the fluorescent probe Mg-Green^TM^. ATP consumption was measured with the addition of saturated antimycin A and oligomycin to block ATP production. P:O ratios were then calculated as ATP produced per oxygen element consumed **(C)**. Data presented as scattered plot of six individuals and mean ± SEM. Statistical difference presented as ^∗^ for *P* < 0.05, tested with two-way ANOVA followed by Turkey’s *post hoc* test.

Two-way ANOVA revealed an interaction between species and acidosis (*F*_1,10_ = 9.01; *P* = 0.01; Figure 5B) as well as a difference between the two species (*F*_1,10_ = 7.8; *P* = 0.02). While ATP production is suppressed with acidosis in *F. varium*, it is increased by ∼3.6-fold in *B. medius*. With a simultaneous decrease in JO_2_ (Figure 5A), this significantly increases the P:O ratio in *B. medius* from 1.14 ± 0.36 at pH 7.25 to 4.95 ± 1.67 at pH 6.65 (*P* = 0.032; Figure 5C). For *F. varium*, at pH 6.65 the JO_2_ trended higher (*P* = 0.06), while the P:O ratio decreased from 1.56 ± 0.65 to -0.59 ± 0.43 (*P* < 0.05).

## Discussion

In the present study, we assessed the *mt* function across a range of pH down to those experienced by hypoxic brain (Katsura et al., 1991, 1992a; Kraut and Madias, 2014; Witt et al., 2017). It is the first study to explore these effects through pH titration and on a range of species with different tolerance to hypoxia, and it revealed significant differences among species that are consistent with species distribution. This differs from studies that test *mt* function with a large variation of pH, as here we titrated unbuffered lactic acid sequentially to modulate pH in order to mimic *in vivo* lactate accumulation and associated pH changes. This includes the progressive nature of pH changes and the duration of changes. Here, we were able to follow *mt* respiration, ΔΨm and pH or JO_2_, Vol_mt_ and pH simultaneously. However, as lactate may modulate the *mt* function and is a possible substrate of neurons (Philp et al., 2005; Chen et al., 2016; Caruso et al., 2017), we divided tissues from single brains to provide a control and reference for comparisons between buffered and unbuffered samples. We show that while intertidal HTS suppress JO_2_ they preserve ΔΨm and ATP production as pH declines. However, the ΔΨm decreased with the suppression of ATP synthesis despite JO_2_ increases in the subtidal species *F. varium*. We contend that with decreasing pH the HTS *mt* may harness the H^+^ accumulation to maintain ΔΨm and ATP synthesis rates.

### Lactate Management of Triplefin Brain *mt*

In the brain, the lactate anion is a putative substrate for aerobic metabolism (reviewed in Barros, 2013; Kane, 2014). While lactate had little influence on JO_2_ for any species in the presence of other *mt* substrates (Figure 2A), lactate and NAD^+^ could sustain OXPHOS at higher rates in the HTS than in *F. varium* (Figure 2B). This indicates a greater capacity for lactate oxidation in HTS, which is likely advantageous post-hypoxia (Dienel, 2012).

### pH Buffering Capacities in the Brain of Triplefin Fish

Under acidosis, cells rely on bicarbonate and non-bicarbonate buffering capacities (Roos and Boron, 1981), and despite the low apparent pH buffering capacity of *mt* (Poburko et al., 2011), *mt* may play a role in the pH regulation when required. We measured the overall buffering capacities of permeabilized brain and isolated *mt*. In both preparations, HSS brains displayed a lower buffering capacity than HTS (Table 1). Although permeabilized tissue buffering differed only marginally (∼5%) across species, isolated *mt* buffering differed by ∼2-fold between *B. medius* and *F. varium*. The *mt* pH buffering contributes to approximately 17% of the cell pH buffering capacity, and while this suggests that some cytosolic components may remain following the permeabilization process, which may significantly buffer pH changes within cells, *mt* also contribute to some pH buffering and protects the *mt* as well and this varies in accordance with hypoxia tolerance.

Comparison of Vol_mt_ also revealed that *mt* from HTS exposed to acidosis swelled by 45% of their initial volume at pH 7.25 (Figure 3). This dilutes matrix solutes, which includes enzymes, substrates and ions including H^+^. A 1.4-fold swelling of the matrix (observed in *F. lapillum*) should mediate an alkalization of approximately 0.15 pH units, preventing excess acidification of the *mt* matrix and would likely assists *mt* pH buffering. In a recent study, acidosis (pH 6.5) mediated *mt* elongation and cristae remodeling (Khacho et al., 2014). While neither pH buffering nor Vol_mt_ were discussed, these observations accord with an increase in Vol_mt_ measured in our study. We note that this is one of the few studies to incorporate estimates of Vol_mt_ into estimates of ΔΨm and it has significant effect. One limitation is that the changes in Vol_mt_ were measured on isolated *mt* and applied for the calculation of ΔΨm in permeabilized tissue. *Mt* exhibit a structural network within cells that is disrupted during the isolation process (Picard et al., 2011). The response of isolated *mt* may therefore differ from the response within the cells. Although the *mt* integrity was similar (RCRs) across preparations (Table 1).

### Acidosis Mediates Contrasted Responses in the Brain *mt* of Hypoxia-Sensitive and Hypoxia-Tolerant Species

Notably in mammalian models, intracellular acidosis has generally been shown to impact *mt* function with some loss of ΔΨm (Tiefenthaler et al., 2001; Bento et al., 2007) and a partial decrease in JO_2_ (Hillered et al., 1984), putatively through CII inhibition (Lemarie et al., 2011). We observed a significant elevation of JO_2_ coincided with a decrease in ΔΨm in *F. varium* (Figure 3). This indicates a loss of OXPHOS efficiency as pH decreases, thereby decreasing ATP synthesis alongside an elevated O_2_ turnover. Moreover, with a substantial drop in ΔΨm, i.e., below -110 mV the ATP_F0-F1_ can reverse and act as a hydrolase, thereby elevating ATP consumption (Chinopoulos et al., 2010).

In contrast, *mt* of the remaining HTS decreased JO_2_ which would appear to be deleterious for OXPHOS (Hillered et al., 1984). However, with these species maintained ΔΨm to moderately low pH (Figure 3). The lesser O_2_ utilization for ΔΨm maintenance suggests that there is either a decrease in proton leak and/or the [H^+^] increase in the media (or cytosol) diffuses into the IMS, may contribute to maintaining the ΔΨm. Notably the permeability of the *mt* outer membrane is high (Cooper, 2000).

### Mitochondria of Hypoxia Tolerant Species May Harness the Extra-*mt* Protons to Maintain Function

The ΔΨm represents the repartition of charge, between the *mt* matrix and the IMS and drives, in part, ATP production (Mitchell, 1961). ΔΨm dissipates with H^+^ transfer into the matrix through three “negative fluxes”; (1) Constitutive leak (not regulated) results from the basal proton diffusion across the inner *mt* membrane; (Jastroch et al., 2010; Divakaruni and Brand, 2011); (2) Inducible leak (regulated), resulting from the proton exchange through proteins (UCPs, ANT, NXHs) (Bernardi, 1999; Halestrap, 2009) that can be modulated by ROS, fatty acids and GDP (Stuart et al., 1999; Jastroch et al., 2010; Masson et al., 2017); and (3) the proton flux used to produce ATP by the F0-F1ATP synthase (Mitchell, 2011). These all act in opposition to the proton flux transferred by the ETS (Mitchell, 2011), or if some of the F0-F1ATP synthases have reversed (Chinopoulos et al., 2010). Work by the ETS (i.e., the positive transfer of H^+^) can be estimated by O_2_ consumption rates and balances these negative fluxes to sustain the ΔΨm. With physiological intracellular pH, the proton gradient (and therefore ΔΨm) that drives ATP production is maintained by the work performed by the ETS.

**FIGURE 6:**
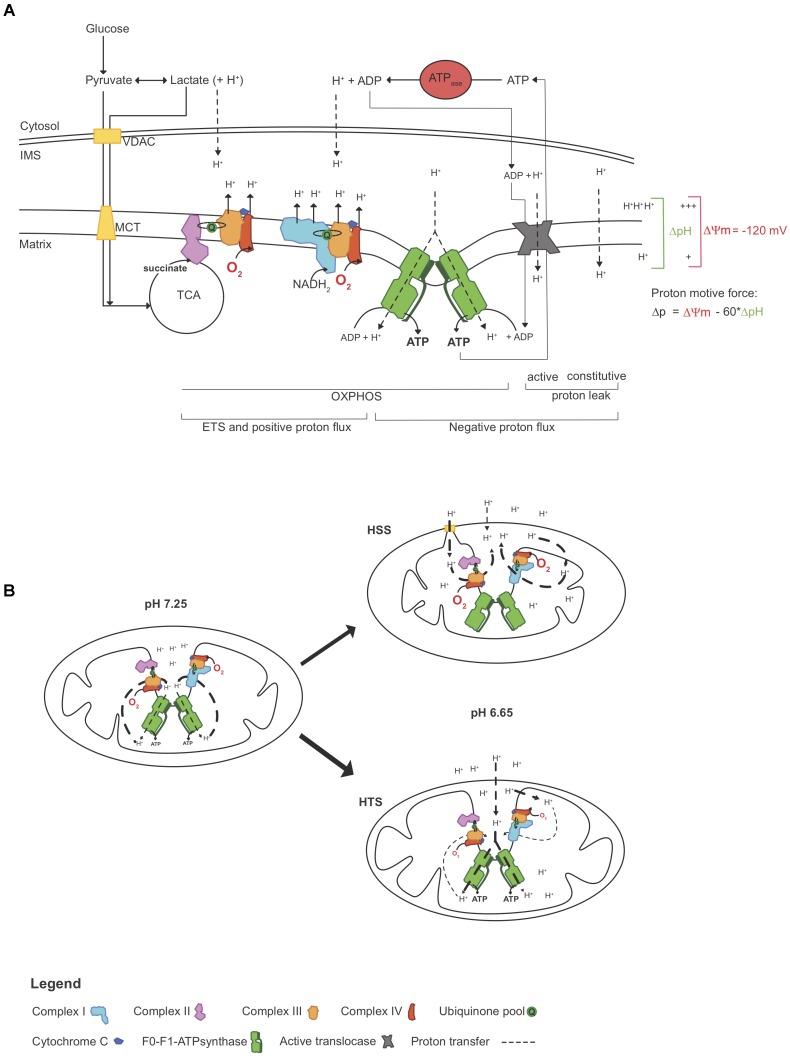
Simplified representation of how acidosis may help to maintain the mitochondrial function. **(A)** ATP production relies on the proton motive force Δp, which in part consists of a proton gradient. In physiological conditions, O_2_ consumption reflects “work” performed by the ETS, which is seen here as proton pumping. At a given Δp, proton pumping equally opposed the combined negative proton fluxes (inward), which includes proton leak (constitutive and active) and “efficient” flux through the F0-F1-ATP synthase. As O_2_ becomes limiting, proton pumping decelerates, decreasing Δp and ATP synthesis. However, as ATP hydrolysis rates surpass synthesis rates, protons accumulating in the cytosol may freely diffuse to the IMS (Cooper, 2000) and these extra-*mt* protons may contribute to Δp, independently ETS “work.” **(B)** In this study, triplefin fish brain mitochondria fish were exposed to gradual acidosis, mediated by the titration of lactic acid. Relative to control (pH 7.25), mitochondria in the subtidal HSS shrank with decreased pH and increased O_2_ consumption rates, but ΔΨm and ATP production decreased. In contrast, intertidal HTS mitochondria swelled and maintained ΔΨm with increased ATP production rates while O_2_ consumption rates were decreased. ΔpH, differential pH; ETS, electron transport system; HSS, hypoxia sensitive species; HTS, hypoxia tolerant species; IMS, inter-membrane space; ΔΨm, mitochondrial membrane potential; MCT, monocarboxylate transporter; OXPHOS, oxidative phosphorylation; Δp, proton motive force; TCA, tricarboxylic acid cycle; VDAC, voltage dependent anion channel.

In 1966, André Jagendorf manipulated the extra-thylakoid pH of chloroplast vesicles to drive ATP synthesis in the dark (Jagendorf and Uribe, 1966), confirming Mitchell’s chemiosmotic hypothesis (Mitchell, 1961) and indicating that ATP synthesis can be mediated with the manipulation of pH changes not generated by the ETS. Here, we sought to assess whereas pH modulation would partially assist ΔΨm and ATP synthesis and if this would influence the work performed by the ETS (illustrated in Figure 6A).

Modulation of the cytosolic pH (i.e., medium pH in permeabilized tissue) mediated an increase of the work performed by the ETS in *mt* of HSS brains (Figure 4), associated with a decrease in ΔΨm (Figure 3C) and ATP synthesis (Figure 5B). With the dogma that acidosis as detrimental to cellular functions, a deleterious effect of acidosis was somewhat expected. However, in brain *mt* of HTS, ΔΨm was maintained despite a decrease in ETS work and ATP production was further increased. This was associated with an elevation of P:O ratios that exceeded 2.7. This indicates that extra-*mt* H^+^ may participate in the proton motive force in phosphorylating *mt* (illustrated in Figure 6B). Such findings are in concordance with a recent study, which showed that in mammalian cortical neurons, mild acidosis (pH 6.5) mediates *mt* remodeling and helps to sustain ATP production regardless of O_2_ levels (Khacho et al., 2014).

## Conclusion

With the increase in glycolytic flux and overall increase in ATP hydrolysis, acidosis mediated by hypoxia generally impairs *mt* function of most vertebrates. In this study, we demonstrate that brain *mt* of hypoxia-tolerant intertidal fish species buffer H^+^ better than the subtidal species *F. varium.* In their natural habitat, as O_2_ dwindles during nocturnal low-tides, intertidal triplefins may turn the problem of acidosis into a temporary solution, and means to sustain ATP synthesis. As opposed to the subtidal *F. varium*, intertidal hypoxia tolerant triplefins appear to take advantage of extra-*mt* protons that helps for maintenance of ΔΨm and ATP production. Acidosis also partially depresses proton pumping by the ETS and resulted in a significant increase in P:O ratio. The increase in *mt* volume also appears to help with ΔΨm maintenance since this dilutes matrix compounds, including protons. We note that a similar process was recently proposed to occur in mammalian cortical neurons (Khacho et al., 2014). The partial suppression of JO_2_ may also slow O_2_ depletion in a hypoxic environment. While the mechanisms underlying the difference between the responses in HSS and HTS are yet to be resolved, these species provide natural strategies that have evolved to support *mt* function in the acidifying brain.

## Availability of Supporting Data

Data supporting the results presented in this article are available at the University of Auckland repository.

## Author Contributions

JD, NB, and AH designed the research. JD performed the research. JD, CH, and AH analyzed the data. JD, CH, NB, NH, GR, and AH wrote the paper.

## Conflict of Interest Statement

The authors declare that the research was conducted in the absence of any commercial or financial relationships that could be construed as a potential conflict of interest.

## Abbreviations

ΔΨm: mitochondrial membrane potential
BLac: buffered lactate
C_add_: additional positive charges
ETS: electron transport system
HSS: hypoxia-sensitive species
HTS: hypoxia-tolerant species
JO_2_: mitochondrial respiration rate
LEAK: leak state respiration
*Mt*: mitochondria
OXPHOS: oxidative phosphorylation state respiration
ULac: unbuffered lactate
Vol_mt_: mitochondrial volume
