# Supplementary material for: Acidosis Maintains the Function of Brain Mitochondria in Hypoxia-Tolerant Triplefin Fish: A Strategy to Survive Acute Hypoxic Exposure?
Source: Front Physiol. 2019 Jan 18;9:1941. doi: 10.3389/fphys.2018.01941 (PMC6346031; doi:10.3389/fphys.2018.01941)
Supplement: Supplementary file 1 [file Data_Sheet_1.docx]

Supplementary Material

Acidosis maintains the function of brain mitochondria in hypoxia-tolerant Triplefin fish: a strategy to survive acute hypoxic exposure?

**Jules B.L. Devaux^1^*, Chris P. Hedges^1^, Nigel Birch^1^, Neil Herbert^2^, Gillian M.C. Renshaw^3^, Anthony J.R. Hickey^1^.**

*** Correspondence**: Jules Devaux, School of Biological Sciences, The University of Auckland, Auckland 1142, New Zealand.

[devauxjules@gmail.com](mailto:devauxjules@gmail.com)

**This Word file includes:** Figures. S1 to S3

Figure S1

Fig. S1. The effect of lactate (left panel) or lactic acid (right panels) on the *mt* function. Respiration rate (JO_2_, A&B), membrane potential (∆Ψm, C) and mitochondrial volume (Vol_mt_, D), were measured at in the presence of saturating mitochondrial (*mt*) substrates and ADP in chambers of oximeters at 20°C (see the material and method section for more details). The difference between the effect of lactic acid and lactate correspond to the effect of acidosis on each *mt* parameter. Results presented as mean (n=7) ± s.e.m.

Figure S2

Fig. S2. Effect of unbuffered lactate on the mitochondrial membrane potential (∆Ψm), without (A) or with (B) the integration of changes in *mt* volume. Estimates of *mt* volume (Vol_mt_) is necessary to calculate ∆Ψm when using cationic dyes. However, Vol_mt_ is dynamic and alters ion repartition and therefore ∆Ψm*.* Here, we simultaneously followed Vol_mt_ and incorporated the relative change to ∆Ψm calculations (see material and method section for more details on the calculation). Results presented as mean (n=7) ± s.e.m.

**Figure S3**

Fig. S3. Mitochondrial respiration mediated by buffered lactate (A) or unbuffered lactate (B). In chambers of respirometer held at 20°C, lactate or lactic acid was titrated on brain homogenates of triplefin fishes in the presence of sufficient ADP but in the absence of other *mt* substrates (i.e. pyruvate, malate, glutamate and succinate). Results presented as mean (n=7) ± s.e.m.
